# How can microfinance institutions successfully navigate a competitive advantage and financial performance? Exploring the role of ambidextrous leadership and intellectual capital

**DOI:** 10.3389/fsoc.2024.1482796

**Published:** 2024-12-02

**Authors:** Syahrul Effendi, Idris Gautama So, Nugroho Juli Setiadi, Gatot Soepriyanto

**Affiliations:** ^1^Management Department, BINUS Business School, Bina Nusantara University, Jakarta, Indonesia; ^2^Accounting Department, School of Accounting, Bina Nusantara University, Jakarta, Indonesia

**Keywords:** ambidextrous leadership, intellectual capital, competitive advantage, financial performance, microfinance industry, Indonesia

## Abstract

The main objective of this study was to investigate how ambidextrous leadership contributes to competitive advantage and financial performance in Indonesia's microfinance institutions (MFIs). A secondary aim was to analyze the moderating effect of intellectual capital on the relationship between ambidextrous leadership and competitive advantage and the mediating role of competitive advantage in the indirect link between ambidextrous leadership and financial performance. Data were collected from 88 firms in the MFI sector through purposive sampling. The Moderation-Mediation (MODMED) procedure was used to assess four proposed relationships. The results indicated that ambidextrous leadership is crucial for achieving competitive advantage, with intellectual capital as a moderator in this relationship. Furthermore, competitive advantage was found to significantly explain financial performance and serve as an intermediary in the connection between ambidextrous leadership and financial performance. This study addresses the existing literature gap by examining ambidextrous leadership's influence on competitive advantage. It also introduces a fresh perspective by suggesting that intellectual capital acts as a boundary condition in the link between ambidextrous leadership and competitive advantage. The findings offer pragmatic insights for organizations, particularly MFIs in Indonesia, to enhance their competitive advantage through effective leadership and strategic management of intellectual resources.

## 1 Introduction

The concept of microfinance emerged in the 1970's when Muhammad Yunus launched a micro-lending initiative in Bangladesh to combat the escalating problem of poverty (Daher and Le Saout, 2013). This innovative program led to the establishment of microfinance institutions (MFIs) across Asia, aimed at addressing widespread poverty and fostering economic and financial growth, emphasizing empowering women, and promoting gender equality. Microfinance has been linked to various positive outcomes, including enhanced educational opportunities for children, improved health conditions, better living standards, and increased job prospects in regions where a significant portion of the population lives in poverty (Iqbal et al., 2019).

Microfinance institutions have traditionally depended on funding from non-profit entities, such as international donors, grants, donations, government assistance, and subsidies, to achieve their poverty alleviation goals (Fadikpe et al., 2022). Consequently, profit-driven organizations, including commercial MFIs, have emerged to achieve financial sustainability through profit-making. However, relying exclusively on these sources is inadequate for the growth and expansion of the microfinance sector (D'Espallier et al., 2013; Fadikpe et al., 2022). This situation has led some policymakers and practitioners to raise concerns about the long-term viability of donor funding. Striking a balance between social and financial performance presents a considerable challenge for MFIs. Many institutions prioritize financial self-sufficiency or profitability, which can lead them away from their social missions. Conversely, MFIs focusing solely on their social objectives may risk their sustainability and profitability (Fadikpe et al., 2022; Green et al., 2023). While social performance is important, MFIs must balance their social missions with financial sustainability. Therefore, analyzing the factors that affect financial performance can assist MFIs in achieving a balance, enabling them to provide social benefits while maintaining their sustainability.

The present study focuses on the determinants of financial performance of microfinance institutions (MFIs) in Indonesia, a developing nation facing significant challenges in poverty alleviation similar to those encountered by other countries such as Pakistan, India, Bangladesh, Thailand, and various African nations (Chauhan, 2021; Hemtanon and Gan, 2020; Khan A. A. et al., 2021; Remer and Kattilakoski, 2021). A critical barrier for the poor in escaping poverty is the lack of access to essential financial services and funding. MFIs can serve as a vital tool in alleviating poverty for populations with limited access to formal banking networks, dispersed geographical locations, inadequate collateral, and low financial literacy. According to the Indonesian Financial Services Authority (OJK), ~203 million Indonesians lack access to financial services, highlighting the crucial role of MFIs in the country. However, like any other nation, MFIs in Indonesia encounter various challenges. These include limited funding, issues related to management quality (such as risk management), and a lack of skills and human resource capacity (Saputra, 2024). Hence, addressing these challenges is essential for enhancing the financial performance of MFIs and, consequently, their ability to contribute to poverty alleviation in Indonesia.

Previous studies have thoroughly investigated various factors that influence the financial performance of microfinance institutions. Key determinants identified include board gender diversity (Ali et al., 2023; Sarpong-Danquah et al., 2023), ownership structure (Khan A. et al., 2021), risk management practices (Ali et al., 2023; Mutamimah et al., 2022), financial support mechanisms (Adusei and Adeleye, 2024), and capital structure (Dabi et al., 2023). A meta-analysis by Hermes and Hudon (2019) highlighted that the most significant determinants discussed in the literature include MFI characteristics (such as size, age, and type of organization), funding sources, the quality of organizational governance, and the external context of MFIs, which encompasses macroeconomic, institutional, and political conditions. However, the evidence surrounding these factors often varies considerably depending on the specific country's context (Hermes and Hudon, 2019). This highlights the importance of conducting context-specific studies to understand better the unique determinants of financial performance in different settings.

The inconclusive findings regarding the determinants of financial performance in microfinance institutions highlight the need for further empirical research. Therefore, this study aims to contribute to the limited existing discourse. Adopting a resource-based perspective (Barney, 1991), leadership and intellectual capital are two critical factors influencing organizational competitiveness and performance (Banmairuroy et al., 2022; Çaǧlıyan et al., 2022; Cantele and Zardini, 2018; Kamukama et al., 2017; Mahdi and Nassar, 2021; Saeidi et al., 2015; Suryantini et al., 2023a,b; Zahid et al., 2021). In addition to exploring the direct impact of ambidextrous leadership on the competitive advantage and financial performance of Indonesian MFIs, the study also investigates whether the intellectual capital of these institutions moderates the relationship between ambidextrous leadership and competitive advantage.

This study makes two significant contributions to the current literature. First, previous research has highlighted the crucial role of ambidextrous leaders in promoting innovation, agility, and overall performance (Ansah et al., 2022; Gerlach et al., 2020; Gouda and Tiwari, 2024; Jiang et al., 2023; Rojas-Córdova et al., 2023; Zacher and Rosing, 2015; Zhang and Suntrayuth, 2024), there has not been a focused investigation of this type of leadership specifically within the context of microfinance institutions. Most prior studies have examined ambidextrous leadership in sectors such as small and medium-sized enterprises (SMEs; Rojas-Córdova et al., 2023), technology and manufacturing industries (Gerlach et al., 2020; Jiang et al., 2023; Zacher and Rosing, 2015; Zhang and Suntrayuth, 2024), and banking (Ansah et al., 2022). Therefore, this study enhances practical knowledge regarding the effectiveness of ambidextrous leadership in microfinance institutions.

**Figure 1:**
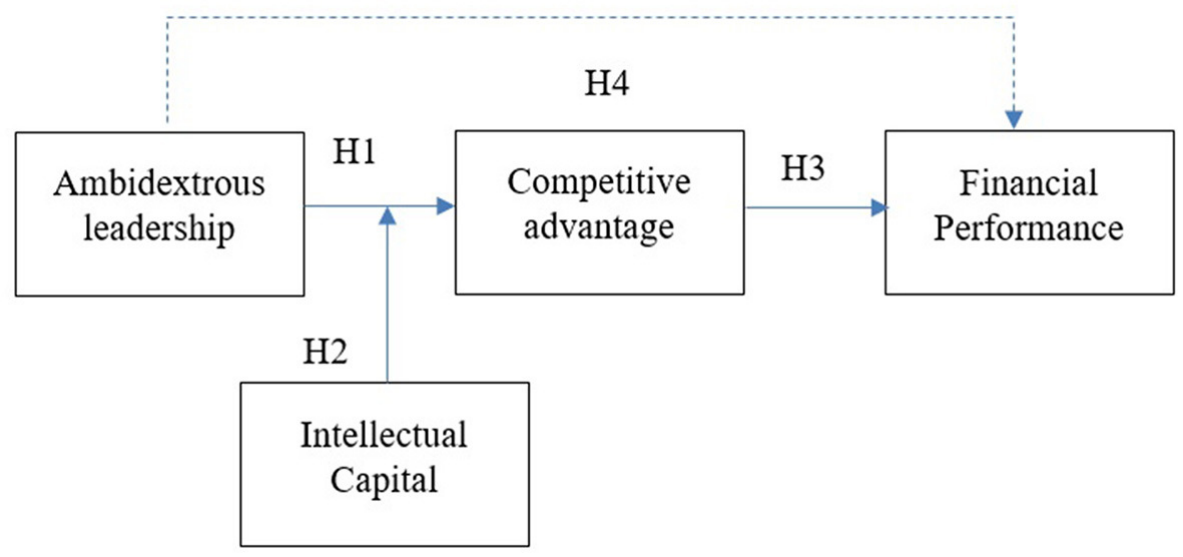
Research model.

Secondly, this study presents a complex model of the relationship between ambidextrous leadership, competitive advantage, intellectual capital, and financial performance. Previous research has emphasized the connections between competitive advantage, intellectual capital, and performance (Çaǧlıyan et al., 2022; Jain et al., 2017; Kim et al., 2018; Lyu et al., 2024; Suryantini et al., 2023a,b; Yang et al., 2018; Zahid et al., 2021). Unlike earlier studies, we introduce intellectual capital as a boundary condition in the relationship between ambidextrous leadership and competitive advantage. This study shifts the perspective by proposing that intellectual capital is also a vital factor in enhancing the effectiveness of ambidextrous leadership, thereby adding a new dimension to our understanding of how intellectual capital functions within organizations. Furthermore, the study addresses a call from Rosing and Zacher (2023) to consider contextual factors when assessing the outcomes of ambidextrous leadership (see Figure 1). The findings also provide practical insights for organizations, particularly microfinance institutions in Indonesia, on strengthening their competitive advantage through ambidextrous leadership and the strategic management of intellectual resources.

### 1.1 Ambidextrous leaders and competitiveness advantage

Ambidextrous leadership refers to the capacity of leaders to effectively manage and blend opposing yet complementary leadership behaviors to improve organizational performance by enhancing their interactive effects using sanctioning errors through closing leader behavior and simultaneously tolerating them through opening leader behavior (Rosing et al., 2011). This unique leadership style is characterized as ambidextrous leadership (Jia et al., 2022; Miron-Spektor et al., 2018). At the micro level, ambidexterity—which includes both exploration and exploitation (Mueller et al., 2020)—empowers organizations to adapt to change and is recognized as a vital dynamic capability necessary for ensuring both current and future sustainability. The capacity to exhibit ambidextrous leadership, which involves nurturing ambidexterity in followers (Rosing et al., 2011), is crucial because exploration requires increasing variance, seeking and adapting to alternatives, taking risks, and engaging in experimentation. Conversely, exploitation demands reducing variance, following established rules, avoiding risks, and ensuring organizational alignment. This dual approach is crucial in today's complex and dynamic business landscape, where leaders must find a balance between control, accountability, innovation, and flexibility (Gerlach et al., 2020; Jiang et al., 2023; Zacher and Rosing, 2015).

The relationship between ambidextrous leadership and competitive advantage is still empirically limited; however, the two are related for several reasons. First, ambidextrous leaders cultivate a culture of innovation by encouraging risk-taking and experimentation (Gerlach et al., 2020; Kung et al., 2020; Rosing et al., 2010; Usman et al., 2022; Wang et al., 2021; Yang et al., 2023; Zacher and Rosing, 2015). They foster flexibility and empower their subordinates (Gerlach et al., 2020; Wang et al., 2022) to generate new ideas and solutions, which can result in the creation of unique products or services that distinguish the organization from its competitors. Second, ambidextrous leadership facilitates continuous organizational learning through exploratory and exploitative practices (Duc et al., 2020; Gerlach et al., 2020). Leaders who promote knowledge-sharing and collaboration contribute to establishing a learning organization that can adapt and evolve (Suryani et al., 2022), which is vital for sustaining a competitive advantage. Finally, ambidextrous leaders are skilled at balancing the dual demands of innovation and pursuing new opportunities (exploration) while refining and optimizing existing processes (exploitation). This dual focus allows organizations to effectively respond to changing market conditions and maximize their resources and capabilities. Organizations enhance their agility by harmonizing these two aspects and contributing to sustained competitive advantage. Therefore, the proposed hypothesis is,

**H1:** Ambidextrous leaders are significantly related to competitive advantage.

### 1.2 The role of intellectual capital

The literature offers a prevalent definition of intellectual capital, characterized by various authors as “packaged useful knowledge” (Stewart, 1997) that can be translated into firm value (Edvinsson and Malone, 1997; Zigan et al., 2007). These definitions underscore intellectual capital as a vital resource that merges practical knowledge with the potential to generate value for the firm. Furthermore, intellectual capital is acknowledged as an intangible yet valuable asset, establishing a robust competitive advantage that influences company performance (Dabić et al., 2019; Kamukama et al., 2011; Rehman et al., 2022; Todericiu and Stăniţ, 2015).

From a resource-based perspective (Barney, 1991), intellectual capital—which includes human, structural, and relational capital—is essential for enhancing the effectiveness of ambidextrous leadership. Firstly, human capital is key to organizational success and competitive advantage (Cisneros et al., 2020; Singh et al., 2022). Skilled and knowledgeable employees are key factors that help companies produce unique products and services, and these conditions can strengthen the innovative environment fostered by ambidextrous leaders. Moreover, in an environment that encourages exploring and exploiting new ideas promoted by ambidextrous leaders, these employees can collaborate more effectively, share knowledge, and drive organizational performance initiatives. This engagement not only supports the goals of ambidextrous leadership but also contributes to the overall competitive advantage of the organization by fostering a culture of continuous improvement and innovation.

Second, structural capital is crucial in the innovation process. Strong organizational structures and systems provide essential support for the initiatives of ambidextrous leaders, encouraging knowledge sharing and collaboration. Companies characterized by an innovative development model—those capable of producing innovative products and responding quickly to ongoing market changes—tend to have a greater share of structural capital compared to those with a traditional development model (García Castro et al., 2021; Shchepkina et al., 2022). This structural foundation enhances the effectiveness of leadership in achieving competitive outcomes.

Lastly, relational capital is vital. Strong relationships with stakeholders, such as customers and partners, can provide valuable insights and resources that support innovation efforts. Ambidextrous leaders who effectively leverage these relationships can strengthen their organization's competitive position further. Thus, this study posits that a high intellectual capital level can amplify ambidextrous leadership's positive effects on competitive advantage.

**H2:** Intellectual capital moderates the relationship between ambidextrous leadership and competitive advantage, such that the positive influence of ambidextrous leadership on competitive advantage is stronger in organizations with high levels of intellectual capital.

### 1.3 Competitiveness advantage and financial performance

In today's competitive environment, having a competitive advantage is essential for ensuring organizational sustainability and performance (Çaǧlıyan et al., 2022; Cantele and Zardini, 2018; Carmeli, 2004; Saeidi et al., 2015; Zahid et al., 2021). Carmeli (2004) emphasizes that competitive advantage is determined by a company's unique attributes that are difficult to imitate and can be maintained over time. This distinct positioning allows organizations to outpace their competitors and achieve superior business results. Hence, there is no doubt that competitive advantage is crucial for a company's overall performance.

The present study also proposes that competitive advantage mediates the relationship between ambidextrous leadership and financial performance based on the following arguments: first, leaders who exhibit ambidextrous leadership can balance exploration (innovation, new ideas) and exploitation (efficiency, optimization of existing processes). Through ambidextrous leadership, organizations can develop unique capabilities and resources that set them apart from competitors. This behavior may include fostering a culture of innovation, enhancing employee engagement, and optimizing operational efficiencies. As a result, the organization gains a competitive edge in the market (Gerlach et al., 2020; Kung et al., 2020; Rosing et al., 2010; Usman et al., 2022; Wang et al., 2021; Yang et al., 2023; Zacher and Rosing, 2015). Secondly, competitive advantage leads to improved financial performance by enabling organizations to achieve higher sales, reduce costs, and increase market share. When an organization is perceived as superior in its offerings or operational efficiency, it can command better pricing, attract more customers, and ultimately enhance profitability (Çaǧlıyan et al., 2022; Cantele and Zardini, 2018; Carmeli, 2004; Saeidi et al., 2015; Zahid et al., 2021). In this context, competitive advantage serves as a mediator by translating the effects of ambidextrous leadership into tangible financial outcomes. The effectiveness of ambidextrous leadership in driving financial performance is thus contingent upon the extent to which it fosters competitive advantages. Furthermore, previous studies have also highlighted the role of competitive advantage as an intermediary factor between corporate social responsibility and financial performance (Jain et al., 2017; Kim et al., 2018; Zahid et al., 2021), as well as between organizational innovativeness and firm performance (Çaǧlıyan et al., 2022), and enterprise risk management practices and firm performance (Yang et al., 2018). Hence, we have a competitive advantage in this study, which we propose as a mediator of the relationship between ambidextrous leaders and financial performance.

**H3:** Competitive advantage and financial performance are positively related.
**H4:** Competitive advantage mediates the relationship between ambidextrous leadership and financial performance.

## 2 Methodology

### 2.1 Participants and procedures

The target sample for this study comprised all microfinance institutions (MFIs) registered with the Indonesian Financial Services Authority. This study focuses on samples at the managerial level for two key reasons. First, managers are typically involved in their organization's decision-making processes and strategic planning. Their insights and experiences are essential for understanding the dynamics of ambidextrous leadership, competitive advantage, and financial performance to ensure that the data collected is directly relevant to its research objectives. Second, managers often have access to sensitive and strategic information that may not be available to lower-level employees. Hence, selecting respondents in managerial positions ensures that the study captures relevant, informed, and strategic insights critical for understanding the complex relationships between ambidextrous leadership, competitive advantage, and financial performance in the microfinance institutions area.

Initially, the researchers invited all 245 management representatives from the registered MFIs to participate; however, only 88 agreed to participate voluntarily (35.92% of all MFIs). Participation was voluntary and uncompensated, with strict measures in place to ensure the anonymity of participants throughout the data collection and analysis process. By completing the questionnaire, participants provided their informed consent. The first page of the questionnaire included a cover letter outlining the study's objectives, data handling procedures, anonymity guarantees, and instructions for filling out the questionnaire.

Most respondents were between 40 and 49 (39.4%), followed by the 30–39 age group at 23.1% and those over 50 years at 22.1%. Regarding educational qualifications, 56.7% of respondents held a Bachelor's degree, while 12.5% had earned a Master's degree. About work experience, 17% had been employed for over 5 years, 39.4% had worked for 3–5 years, and 17.3% had <3 years of experience. The participants held different positions, with the majority being Managers (34.6%), followed by main directors (29.8%), treasurers/secretaries (15.4%), and deputy directors (10.6%). This variety of leadership roles provides a thorough understanding of the leadership structure within Indonesian MFIs.

### 2.2 Measurement

All questionnaires were initially created in English and subsequently translated into Indonesian to meet the participant's language preferences. To ensure the measures were equivalent, we implemented a back-translation procedure using the guidelines established by Brislin (1980).

This study evaluated ambidextrous leadership using a set of 14 items based on the framework established by Rosing et al. (2011). While the original scale comprises explorative and exploitative dimensions, some research has opted for a single-factor measurement model (Laser, 2022). This study used exploratory factor analysis (EFA) to reassess the factor structure, resulting in a unidimensional construct of eight items consistent with previous studies (Isichei et al., 2022; Tang and Wei, 2023; Wu et al., 2020). According to Laser (2022), this single-factor model is prevalent because it necessitates switching between behaviors that promote exploration and exploitation; the criteria in this category are always applicable to both aspects. Consequently, there is no compelling reason to differentiate between them, and all top executives must meet these criteria, regardless of their primary focus on exploration or exploitation within the organization. This study identified eight items, such as “encouraging experimentation with different ideas” alongside “monitoring and controlling,” pertinent to exploration and exploitation, particularly in the microfinance sector. Participants rated their responses on a five-point Likert scale, ranging from “never” (1) to “always” (5).

Competitive advantage was evaluated with four items based on Jain et al. (2017). Examples are “Our firm has access to resources at competitive rates” and “Our firm has been able to increase market share in the last few years.” Respondents were asked to provide a rating on a five-point Likert scale, ranging from “low” (1) to “high” (5). Financial performance was measured by examining the growth in capital, liabilities, and non-performing loans, adapted from Kamukama et al. (2017). Respondents were asked to provide a rating on a five-point Likert scale, ranging from “low” (1) to “high” (5).

The measuring intellectual capital is based on a content analysis index approach derived from interviews with the managers. The items used in the analysis are adapted from previous research (Keter et al., 2024; Sardo and Serrasqueiro, 2017) and cover three dimensions: structural capital (SC), relational capital (RC), and human capital (HC), with a total of 19 adjusted items. Per the senior manager's statement, each item is typically scored on a scale of 1–5. Moreover, the overall intellectual capital (IC) for the company is calculated as follows:


$$\begin{array}{l} IC=\frac{d}{M}\;x\;100\% \end{array}$$

Where d is the total score of the manager's answers, and M denotes the maximum score of all IC items (19 x 5 = 95).

The results presented in Table 1 indicate that the variables examined demonstrate convergent validity, as all items achieved factor loadings above the accepted threshold of 0.50 and average variance explained (AVE) > 0.50 (Hair et al., 2019). Additionally, all scales utilized in the study meet the reliability criteria, with a minimum Cronbach alpha threshold of 0.70 (Nunnally and Bernstein, 1994).

**Table 1:** Measurement evaluation.

**Variable**	**Factor loading**	**α**	**AVE**
Ambidextrous leadership	0.81–0.88	0.93	0.72
Intellectual capital	0.65–0.80	0.89	0.52
Competitiveness advantage	0.73–0.79	0.85	0.58
Financial performance	0.59–0.81	0.73	0.52

#### 2.2.1 Analysis strategy

To test the hypotheses in our study, we employed moderation and mediation analysis techniques by the procedures using SPSS macro developed by Hayes (2017) to estimate both mediation and moderated mediation models. Furthermore, we conducted a bootstrapping analysis to evaluate the significance of the effects, generating 5,000 bootstrapped samples to calculate bias-corrected confidence intervals for the estimated effects. *Bootstrapping* is a statistical resampling method that enhances inference robustness and yields more accurate indirect and moderated effects estimates in mediation and moderation analyses (Hayes, 2017).

## 3 Results

### 3.1 Descriptive statistics and correlation

The average scores listed in Table 2 demonstrate different perceptions of important organizational factors. Ambidextrous leadership has a moderate score of 3.69, indicating a reasonable implementation of practices that balance exploration and exploitation. Competitiveness advantage is rated at 3.74, showing effective strategies enhancing the organization's market position. Intellectual capital is recognized at 83.17%, showing an understanding of the value of intellectual resources. On the other hand, financial performance has a low rating of 1.98, signaling significant concerns about the organization's capital, liabilities, and non-performing loans, highlighting the urgent need for improvement in this area.

**Table 2:** Descriptive statistics and correlation.

**No**	**Variable**	**Mean**	**S.D**	**1**	**2**	**3**	**4**
1	Ambidextrous leadership	3.69	0.87	1			
2	Competitiveness advantage	3.74	0.76	0.590^**^	1		
3	Intellectual capital	83.17	8.96	0.178	0.090	1	
4	Financial performance	1.98	0.81	0.579^**^	0.587^**^	0.335^**^	1

^**^p < 0.01. or indicates statistical significance at the 1% level.

The correlation analysis presented in Table 2 reveals several significant relationships among the variables: ambidextrous leadership is positively correlated with both competitiveness advantage (*r* = 0.590, *p*-value < 0.01) and financial performance (*r* = 0.579, *p*-value < 0.05), indicating that effective leadership practices contribute to enhanced competitive positioning and better financial outcomes; however, no significant correlation is found between ambidextrous leadership and intellectual capital. Additionally, competitiveness advantage shows a strong positive correlation with financial performance (*r* = 0.587, *p*-value < 0.01), suggesting that a competitive edge translates into improved financial results. Lastly, intellectual capital is positively associated with financial performance (*r* = 0.335, *p*-value < 0.05), indicating that the value of intellectual resources also enhances financial success.

### 3.2 Hypothesis testing

**Figure 2:**
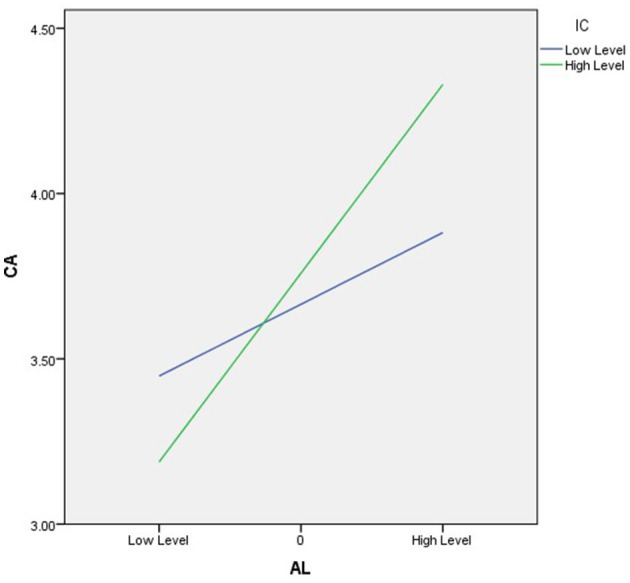
The moderating effect of intellectual capital on the relationship between ambidextrous leadership and competitive advantage.

Hypothesis 1 suggests that having ambidextrous leaders is linked to a competitive advantage, and the findings support this hypothesis. A significant positive relationship was found (β = 0.453, *p* < 0.01), as shown in Table 3. Hypothesis 2 suggests that intellectual capital impacts the relationship between ambidextrous leadership and competitive advantage, and the results confirm this hypothesis. The PROCESS analysis shows that the interaction between ambidextrous leadership and intellectual capital is positively and significantly associated with financial performance. This study used a bias-corrected construct with 5,000 bootstrapped samples to determine confidence intervals (CIs) for all significance tests (Cahyadi et al., 2021; Xie et al., 2018). Table 4 and Figure 2 show that the direct relationship between ambidextrous leadership and competitive advantage was significant for both low and high levels of intellectual capital in the control group, with values of (β = 0.246; *p* < 0.05) and (β = 0.656; *p* < 0.01), respectively. As a result, both H1 and H2 are supported.

**Table 3:** Hypothesis testing results.

	**Coeff**	**Se**	***t***	***p***	**LLCI**	**ULCI**
**Model 1, competitiveness advantage as outcome variable**						
Ambidextrous leadership	0.453	0.077	5.877	0.000	0.300	0.606
Interaction	0.023	0.008	2.929	0.000	0.007	0.038
**Model 2, financial performance as outcome variable**						
Ambidextrous leadership	0.331	0.094	3.510	0.001	0.143	0.518
Competitiveness advantage	0.402	0.108	3.706	0.000	0.186	0.618

IC, intellectual capital; LLCI, low-level of confidence interval; ULCI, upper level of confidence interval.

**Table 4:** The conditional effect of moderated and mediation analysis.

	**Coeff**	**Se**	***t***	***p***	**LLCI**	**ULCI**
Indirect effect	0.182	0.041	-	-	0.104	0.266
**Conditional effect of the ambidextrous leadership at value of intellectual capital as moderator**						
−1 SD (low level of IC)	0.249	0.117	2.132	0.036	0.017	0.482
+1 SD (high level of IC)	0.656	0.088	7.421	0.000	0.480	0.832

IC, intellectual capital; LLCI, low-level of confidence interval; ULCI, upper level of confidence interval.

Hypothesis 3 posits a link between competitive advantage and financial performance, and the results reveal a significant relationship (β = 0.402, *p* < 0.01). Furthermore, Hypothesis 4 suggests that competitive advantage mediates the relationship between ambidextrous leadership and financial performance, as illustrated in Table 4. The findings corroborate hypothesis 4, demonstrating that competitive advantage significantly mediates the connection between ambidextrous leadership and financial performance, with a mediation effect of (β = 0.182) and confidence intervals of LLCI = 0.104 and ULCI = 0.266.

## 4 Discussion

This study seeks to provide empirical evidence for the hypothesis regarding the influence of ambidextrous leadership on improving competitive advantage and financial performance within microfinance institutions in Indonesia. The results indicate that ambidextrous leaders play a significant role in fostering competitive advantage, with intellectual capital moderating in this relationship. Furthermore, competitive advantage is identified as a key determinant of financial performance, serving as an intermediary between ambidextrous leadership and financial performance.

The present study introduces a novel model that clarifies the relationship between ambidextrous leadership, competitive advantage and financial performance. This research highlights the significant role that ambidextrous leadership plays in establishing competitive advantage, providing initial empirical evidence in this area. It builds upon previous studies that have primarily examined the relationship between ambidextrous leadership and various outcomes, such as innovation, agility, and overall performance (Gerlach et al., 2020; Gouda and Tiwari, 2024; Jiang et al., 2023; Zacher and Rosing, 2015; Zhang and Suntrayuth, 2024). Consequently, this study offers foundational empirical support for the direct link between ambidextrous leadership and competitive advantage, particularly within the context of microfinance institutions. This contribution enriches the existing literature by demonstrating how ambidextrous leadership enhances competitive advantage, providing a more comprehensive understanding of the implications of ambidextrous leadership styles in microfinance organizational settings.

In the same vein, the present study introduces the concept of intellectual capital as a boundary condition, meaning that it influences how ambidextrous leadership affects competitive advantage. The research findings indicate that the positive effects of ambidextrous leadership on competitive advantage are stronger when an organization has high levels of intellectual capital. These results suggest that organizations with high intellectual resources can leverage ambidextrous leadership more effectively to gain a competitive edge. Traditionally, intellectual capital has been viewed mainly as a precursor or contributing factor to competitive advantage (Dabić et al., 2019; Kamukama et al., 2011; Rehman et al., 2022; Todericiu and Stăniţ, 2015). The present study shifts that perspective by showing that intellectual capital also plays a critical role in enhancing the effectiveness of ambidextrous leadership, thus adding a new layer to the understanding of how intellectual capital functions within organizations. Moreover, the study also addresses a call from Rosing and Zacher (2023) to consider contextual factors when examining the outcomes of ambidextrous leadership.

Finally, the present study uncovers a model that illustrates the indirect relationship between ambidextrous leadership and financial performance, mediated by competitive advantage. While previous research (Suryantini et al., 2023a,b) identified intellectual capital as a key predictor of performance and sustainable competitive advantage, this study introduces ambidextrous leadership as an additional predictor for both competitive advantage and financial performance. Furthermore, whereas Suryantini's work focused on enhancing the understanding of small and medium-sized enterprise (SME) development, this research shifts the emphasis to microfinance institutions, marking a significant progression in the field. This transition expands the scope of inquiry and underscores the importance of ambidextrous leadership in a different organizational context, thereby enriching our understanding of how various leadership styles can impact competitive dynamics and financial outcomes within the microfinance sector.

### 4.1 Practical implications

The study highlights the important role of ambidextrous leadership in microfinance institutions and how it impacts competitive advantage and financial performance. The findings emphasize that ambidextrous leadership, which involves balancing exploration (innovation and new opportunities) and exploitation (efficiency and optimization of existing resources), is crucial for organizations in dynamic and competitive environments like microfinance. The research suggests that ambidextrous leadership directly contributes to developing competitive advantage, enabling leaders to differentiate their institutions and enhance competitiveness. Furthermore, the study shows that the competitive advantage gained through ambidextrous leadership significantly improves the financial performance of microfinance institutions. As a result, cultivating ambidextrous leadership can help organizations improve their competitive positioning and achieve better financial outcomes. The study also proposes that the ambidextrous leadership model can be a framework for microfinance institutions to develop effective competitiveness strategies. It emphasizes the importance of adopting an ambidextrous leadership style and prioritizing the development of competitive advantages for better financial performance. Finally, the research suggests that future studies explore how specific ambidextrous leadership behaviors can be cultivated and measured within microfinance institutions and examine the interplay between different types of competitive advantages and financial performance for deeper insights into effective management practices in this sector. Hence, managers should actively adopt ambidextrous leadership styles that balance exploration and exploitation, encouraging innovation and new ideas while optimizing existing processes and resources. Training programs and leadership development initiatives can be implemented to cultivate these skills among current and future leaders.

Intellectual capital plays a crucial role in enhancing the effectiveness of ambidextrous leaders within organizations. Therefore, organizations need to prioritize investments in intellectual capital, which encompasses human capital (such as the skills and knowledge of employees), structural capital (comprising processes, systems, and culture), and relational capital (including relationships with stakeholders). To support ambidextrous leadership, organizations can create a more favorable environment to maximize their competitive advantage. Organizations should implement comprehensive training and development programs to improve employees' skills and knowledge; this not only enhances individual performance but also strengthens the overall intellectual capital of the organization. Additionally, management can establish strategic partnerships with educational institutions, research organizations, and other entities to enhance the organization's intellectual capital. Collaborations provide access to new knowledge, skills, and resources that can be utilized to support ambidextrous leadership.

### 4.2 Limitations

The study has some limitations that point to opportunities for future research. First, the research is limited by the companies' young age, ranging from 2 to 5 years. The microfinance institutions in Indonesia are still relatively new, having been operational for only 5 years. Consequently, the results may only provide an early snapshot of the microfinance landscape, indicating a need for further studies to validate and expand upon these findings. Secondly, the study's use of a cross-sectional design and a single data source limits the ability to make causal claims and may introduce common method bias (Podsakoff et al., 2012). Future research should consider a longitudinal approach and use a mix of primary and secondary data sources to more accurately assess the financial performance of microfinance institutions. Additionally, the study is confined to a specific country and geographical area, which restricts the generalizability of the findings to other regions and countries. Therefore, future research should investigate how ambidextrous leadership behaviors influence competitive advantage and financial performance in diverse contexts and locations.

## 5 Conclusions

The current study aims to investigate the impact of ambidextrous leadership on the competitive advantage and financial performance of microfinance institutions in Indonesia. Additionally, we examine the moderating role of intellectual capital in the relationship between ambidextrous leadership and competitive advantage and the mediating role of competitive advantage in the connection between ambidextrous leadership and financial performance. The findings reveal a direct relationship between ambidextrous leadership and competitive advantage, with intellectual capital enhancing the strength of this relationship. Furthermore, the study establishes competitive advantage as an antecedent of financial performance and an intermediary role in the relationship between ambidextrous leadership and financial performance. These insights are valuable for policymakers in Indonesian microfinance institutions, emphasizing the need to leverage internal resources—such as leadership and intellectual capital—to improve competitive advantage and financial performance, thereby enhancing the sector's overall contribution to the economy and society.

## Data Availability

The raw data supporting the conclusions of this article will be made available by the authors, without undue reservation.

## References

[B1] Adusei M. Adeleye N. (2024). Start-up microenterprise financing and financial performance of microfinance institutions. J. Small Bus. Entrepr. 36, 183–206. 10.1080/08276331.2020.1842047

[B2] Ali H. Gueyie J.-P. Chrysostome E. V. (2023). Gender, credit risk and performance in sub-saharan African microfinance institutions. J. Afri. Bus. 24, 235–259. 10.1080/15228916.2022.2079275

[B3] Ansah M. O. Addai-Boamah N. Bamfo A. B. Ry-Kottoh L. A. (2022). Organizational ambidexterity and financial performance in the banking industry: evidence from a developing economy. J. Fin. Serv. Market. 27, 250–263. 10.1057/s41264-021-00117-w

[B4] Banmairuroy W. Kritjaroen T. Homsombat W. (2022). The effect of knowledge-oriented leadership and human resource development on sustainable competitive advantage through organizational innovation's component factors: evidence from Thailand 's new S- curve industries. Asia Pacif. Manag. Rev. 27, 200–209. 10.1016/j.apmrv.2021.09.001

[B5] Barney J. (1991). Firm resources and sustained competitive advantage. J. Manag. 17, 99–120. 10.1177/014920639101700108

[B6] Brislin R. W. (1980). “Translation and content analysis of oral and written material,” in Handbook of Cross-Cultural Psychology, 2nd Edn, eds. H. Triandis and J. Berry (Boston, MA: Allyn & Bacon), 389–444.

[B7] Çaǧlıyan V. Attar M. Abdul-Kareem A. (2022). Assessing the mediating effect of sustainable competitive advantage on the relationship between organisational innovativeness and firm performance. Compet. Rev. 32, 618–639. 10.1108/CR-10-2020-0129

[B8] Cahyadi A. Hendryadi H. Mappadang A. (2021). Workplace and classroom incivility and learning engagement: the moderating role of locus of control. Int. J. Educ. Integr. 17:4. 10.1007/s40979-021-00071-z

[B9] Cantele S. Zardini A. (2018). Is sustainability a competitive advantage for small businesses? an empirical analysis of possible mediators in the sustainability-financial performance relationship. J. Clean. Prod. 182, 166–176. 10.1016/j.jclepro.2018.02.016

[B10] Carmeli A. (2004). Strategic human capital and the performance of public sector organizations. Scand. J. Manag. 20, 375–392. 10.1016/j.scaman.2003.11.003

[B11] Chauhan S. (2021). Social and financial efficiency: a study of Indian Microfinance Institutions. IIM Kozhikode Soc. Manag. Rev. 10, 31–43. 10.1177/227797522095331138165859

[B12] Cisneros M. A. I. Perlines F. H. García M. R. (2020). Intellectual capital, organisational performance and competitive advantage. Eur. J. Int. Manag. 14, 976. 10.1504/EJIM.2020.11058535009967

[B13] Dabi R. S. K. Nugraha D. Sari M. (2023). Capital structure, financial performance and sustainability of Microfinance Institutions (MFIs) in Ghana. Cogent Econ. Fin. 11:13. 10.1080/23322039.2023.2230013

[B14] Dabić M. Lažnjak J. Smallbone D. Švarc J. (2019). Intellectual capital, organisational climate, innovation culture, and SME performance. J. Small Bus. Enterpr. Dev. 26, 522–544. 10.1108/JSBED-04-2018-011739304265

[B15] Daher L. Le Saout E. (2013). Microfinance and financial performance. Strat. Change 22, 31–45. 10.1002/jsc.1920

[B16] D'Espallier B. Hudon M. Szafarz A. (2013). Unsubsidized microfinance institutions. Econ. Lett. 120, 174–176. 10.1016/j.econlet.2013.04.021

[B17] Duc L. A. Tho N. D. Nakandala D. Lan Y.-C. (2020). Team innovation in retail services: the role of ambidextrous leadership and team learning. Serv. Bus. 14, 167–186. 10.1007/s11628-020-00412-x

[B18] Edvinsson L. Malone M. (1997). Intellectual Capital: Realizing Your Company's True Value by Finding Its Hidden Brain-Power. New York, NY: Harper-Collins.

[B19] Fadikpe A. A. A. Danquah R. Aidoo M. Chomen D. A. Yankey R. Dongmei X. (2022). Linkages between social and financial performance: evidence from Sub-Saharan Africa microfinance institutions. PLoS ONE 17, 1–23. 10.1371/journal.pone.0261326 35231026 PMC9090451

[B20] García Castro J. P. Duque Ramírez D. F. Moscoso Escobar J. (2021). The relationship between intellectual capital and financial performance in Colombian listed banking entities. Asia Pacif. Manag. Rev. 26, 237–247. 10.1016/j.apmrv.2021.03.002

[B21] Gerlach F. Hundeling M. Rosing K. (2020). Ambidextrous leadership and innovation performance: a longitudinal study. Leaders. Org. Dev. J. 41, 383–398. 10.1108/LODJ-07-2019-0321

[B22] Gouda G. K. Tiwari B. (2024). Ambidextrous leadership: a distinct pathway to build talent agility and engagement. Hum. Resour. Dev. Int. 27, 133–141. 10.1080/13678868.2022.2163101

[B23] Green W. N. Chhom T. Mony R. Estes J. (2023). The underside of microfinance: performance indicators and informal debt in Cambodia. Dev. Change 54, 780–803. 10.1111/dech.12778

[B24] Hair J. Black W. Babin B. Anderson R. (2019). Multivariate Data Analysis, 8th Edn. Hampshire: Cengage Learning.

[B25] Hayes A. F. (2017). Introduction to Mediation, Moderation, and Conditional Process Analysis: A Regression-Based Approach. New York, NY: Guilford Publications.

[B26] Hemtanon W. Gan C. (2020). Microfinance participation in Thailand. J. Risk Fin. Manag. 13:122. 10.3390/jrfm13060122

[B27] Hermes N. Hudon M. (2019). Determinants of the performance of microfinance institutions: a systematic review. Contempor. Top. Fin. 10, 297–330. 10.1002/9781119565178.ch1036571966

[B28] Iqbal S. Nawaz A. Ehsan S. (2019). Financial performance and corporate governance in microfinance: evidence from Asia. J. Asian Econ. 60, 1–13. 10.1016/j.asieco.2018.10.002

[B29] Isichei E. E. Aminu A. A. Chukwu B. I. Ike N. M. Agbaeze K. E. Anthony I. (2022). Linking ambidextrous leadership and small and medium scale enterprises export performance. South Afri. J. Bus. Manag. 53, 1–14. 10.4102/sajbm.v53i1.2791

[B30] Jain P. Vyas V. Roy A. (2017). Exploring the mediating role of intellectual capital and competitive advantage on the relation between CSR and financial performance in SMEs. Soc. Responsibil. J. 13, 1–23. 10.1108/SRJ-04-2015-0048

[B31] Jia R. Hu W. Li S. (2022). Ambidextrous leadership and organizational innovation: the importance of knowledge search and strategic flexibility. J. Knowl. Manag. 26, 781–801. 10.1108/JKM-07-2020-0544

[B32] Jiang Y. Asante D. Zhang J. Ampaw E. M. (2023). The influence of ambidextrous leadership on the employee innovative behavior: an empirical study based on Chinese manufacturing enterprises. Curr. Psychol. 42, 9452–9465. 10.1007/s12144-021-02233-1

[B33] Kamukama N. Ahiauzu A. Ntayi J. M. (2011). Competitive advantage: mediator of intellectual capital and performance. J. Intellect. Capit. 12, 152–164. 10.1108/14691931111097953

[B34] Kamukama N. Kyomuhangi D. S. Akisimire R. Orobia L. A. (2017). Competitive advantage: mediator of managerial competence and financial performance of commercial banks in Uganda. Afri. J. Econ. Manag. Stud. 8, 221–234. 10.1108/AJEMS-10-2016-0142

[B35] Keter C. K. S. Cheboi J. Y. Kosgei D. (2024). Financial performance, intellectual capital disclosure and firm value: the winning edge. Cogent Bus. Manag. 11:2302468. 10.1080/23311975.2024.2302468

[B36] Khan A. Ahmad A. Shireen S. (2021). Ownership and performance of microfinance institutions: empirical evidences from India. Cogent Econ. Fin. 9:1930653. 10.1080/23322039.2021.1930653

[B37] Khan A. A. Khan S. U. Fahad S. Ali M. A. S. Khan A. Luo J. (2021). Microfinance and poverty reduction: new evidence from Pakistan. Int. J. Fin. Econ. 26, 4723–4733. 10.1002/ijfe.2038

[B38] Kim K.-H. Kim M. Qian C. (2018). Effects of corporate social responsibility on corporate financial performance: a competitive-action perspective. J. Manag. 44, 1097–1118. 10.1177/0149206315602530

[B39] Kung C.-W. Uen J. F. Lin S.-C. (2020). Ambidextrous leadership and employee innovation in public museums. Chin. Manag. Stud. 14, 995–1014. 10.1108/CMS-05-2018-0523

[B40] Laser J. (2022). Criteria to appraise top executives for ambidextrous leadership. J. Org. Effect. 9, 449–470. 10.1108/JOEPP-06-2020-0094

[B41] Lyu C. Peng C. Yang X. Liu F. (2024). Is ambidextrous learning a driver for SMEs' sustainable competitive advantage in the digital era? an empirical study of SMEs in Nanjing, China. Corpor. Soc. Responsibil. Environ. Manag. 31, 1052–1062. 10.1002/csr.2622

[B42] Mahdi O. R. Nassar I. A. (2021). The business model of sustainable competitive advantage through strategic leadership capabilities and knowledge management processes to overcome COVID-19 pandemic. Sustainability 13:9891. 10.3390/su13179891

[B43] Miron-Spektor E. Ingram A. Keller J. Smith W. K. Lewis M. W. (2018). Microfoundations of organizational paradox: the problem is how we think about the problem. Acad. Manag. J. 61, 26–45. 10.5465/amj.2016.0594

[B44] Mueller J. Renzl B. Will M. G. (2020). Ambidextrous leadership: a meta-review applying static and dynamic multi-level perspectives. Rev. Manag. Sci. 14, 37–59. 10.1007/s11846-018-0297-9

[B45] Mutamimah M. Zaenudin Z. Bin Mislan Cokrohadisumarto W. (2022). Risk management practices of Islamic microfinance institutions to improve their financial performance and sustainability: a study on Baitut Tamwil Muhammadiyah, Indonesia. Qual. Res. Fin. Mark. 14, 679–696. 10.1108/QRFM-06-2021-0099

[B46] Nunnally B. Bernstein I. (1994). Psychometric Theory. Oxford: Oxford University.

[B47] Podsakoff P. M. MacKenzie S. B. Podsakoff N. P. (2012). Sources of method bias in social science research and recommendations on how to control it. Ann. Rev. Psychol. 63, 539–569. 10.1146/annurev-psych-120710-100452 21838546

[B48] Rehman S. U. Bresciani S. Ashfaq K. Alam G. M. (2022). Intellectual capital, knowledge management and competitive advantage: a resource orchestration perspective. J. Knowl. Manag. 26, 1705–1731. 10.1108/JKM-06-2021-0453

[B49] Remer L. Kattilakoski H. (2021). Microfinance institutions' operational self-sufficiency in sub-Saharan Africa: empirical evidence. Int. J. Corpor. Soc. Responsibil. 6:5. 10.1186/s40991-021-00059-5

[B50] Rojas-Córdova C. Pertuze J. A. Williamson A. J. Leatherbee M. (2023). More structure or better social practices? using a contingency lens to address ambidexterity gaps in innovative SMEs. Int. J. Emerg. Mark. 18, 5581–5606. 10.1108/IJOEM-04-2021-0572

[B51] Rosing K. Frese M. Bausch A. (2011). Explaining the heterogeneity of the leadership-innovation relationship: ambidextrous leadership. Leaders. Quart. 22, 956–974. 10.1016/j.leaqua.2011.07.014

[B52] Rosing K. Rosenbusch N. Frese M. (2010). “Ambidextrous leadership in the innovation process,” in Innovation and International Corporate Growth, eds. A. Gerybadze, U. Hommel, H. W. Reiners, and D. Thomaschewski (Berlin, Heidelberg: Springer), 191–204. 10.1007/978-3-642-10823-5_12

[B53] Rosing K. Zacher H. (2023). “Ambidextrous leadership: a review of theoretical developments and empirical evidence,” in Handbook of Organizational Creativity, eds. R. Reiter-Palmon and S. Hunter (Amsterdam: Elsevier), 51–70. 10.1016/B978-0-323-91841-1.00013-0

[B54] Saeidi S. P. Sofian S. Saeidi P. Saeidi S. P. Saaeidi S. A. (2015). How does corporate social responsibility contribute to firm financial performance? the mediating role of competitive advantage, reputation, and customer satisfaction. J. Bus. Res. 68, 341–350. 10.1016/j.jbusres.2014.06.024

[B55] Saputra F. (2024). OJK Beberkan penyebab lembaga keuangan mikro berguguran sepanjang tahun ini [OJK Reveals Reasons Why Microfinance Institutions Have Fallen Throughout This Year]. Kontan.Co.Id. Available at: https://keuangan.kontan.co.id/news/ojk-beberkan-penyebab-lembaga-keuangan-mikro-berguguran-sepanjang-tahun-ini (Retrieved September 15, 2024).

[B56] Sardo F. Serrasqueiro Z. (2017). A European empirical study of the relationship between firms' intellectual capital, financial performance and market value. J. Intellect. Capit. 18, 771–788. 10.1108/JIC-10-2016-0105

[B57] Sarpong-Danquah B. Adusei M. Magnus Frimpong J. (2023). Effect of board gender diversity on the financial performance of microfinance institutions: does judicial efficiency matter? Ann. Publ. Cooperat. Econ. 94, 495–518. 10.1111/apce.12396

[B58] Shchepkina N. Meshkova N. Goigova M. Maisigova L. Tochieva L. (2022). Intellectual capital as a factor in ensuring the competitiveness of the railway transport enterprises. Transport. Res. Proced. 63, 1444–1453. 10.1016/j.trpro.2022.06.155

[B59] Singh R. K. Agrawal S. Modgil S. (2022). Developing human capital 4.0 in emerging economies: an industry 4.0 perspective. Int. J. Manpower 43, 286–309. 10.1108/IJM-03-2021-0159

[B60] Stewart T. A. (1997). Intellectual Capital: The New Wealth of Organizations. New York, NY: Bantam Doubleday Dell Publishing Group.

[B61] Suryani S. Sudrajat B. Hendryadi H. Saihu M. Amalia E. Fathoni M. A. (2022). Development of thriving at work and organizational citizenship behavior through Islamic work ethics and humble leadership. Asian J. Bus. Ethics 22:3. 10.1007/s13520-022-00160-3

[B62] Suryantini N. P. S. Moeljadi M. Aisjah S. Ratnawati K. (2023a). The sustainable competitive advantage of SMES towards intellectual capital: the role of technology adoption and strategic flexibility. Intellect. Econ. 17, 30–56. 10.13165/IE-23-17-1-02

[B63] Suryantini N. P. S. Moeljadi M. Aisjah S. Ratnawati K. (2023b). Enhancing sustainable competitive advantage in SMEs: aligning intellectual capital and business performance model. Qual. Access Success 25:33. 10.47750/QAS/25.198.33

[B64] Tang X. Wei S. (2023). Leading for employees' enterprise system ambidextrous use through contextual ambidexterity: the mediating role of user empowerment and moderating role of leader-member exchange. Intern. Res. 2021:645. 10.1108/INTR-09-2021-0645

[B65] Todericiu R. Stǎniţ A. (2015). Intellectual capital—the key for sustainable competitive advantage for the SME's sector. Proced. Econ. Fin. 27, 676–681. 10.1016/S2212-5671(15)01048-5

[B66] Usman M. Ghani U. Islam Z. U. Gul H. Mahmood K. (2022). Ambidextrous leadership and innovative work behaviors: workplace thriving as a mediator. J. Publ. Affairs 22:2321. 10.1002/pa.2321

[B67] Wang L. Sun Y. Li J. Xu Y. Chen M. Zhu X.. (2022). Effects of ambidextrous leadership on employees' work behavior: the mediating role of psychological empowerment. Front. Psychol. 13:862799. 10.3389/fpsyg.2022.862799 35651581 PMC9150796

[B68] Wang S. Eva N. Newman A. Zhou H. (2021). A double-edged sword: the effects of ambidextrous leadership on follower innovative behaviors. Asia Pacif. J. Manag. 38, 1305–1326. 10.1007/s10490-020-09714-0

[B69] Wu M. Wang R. He P. Estay C. Akram Z. (2020). Examining how ambidextrous leadership relates to affective commitment and workplace deviance behavior of employees: the moderating role of supervisor-subordinate exchange Guanxi. Int. J. Environ. Res. Publ. Health 17:5500. 10.3390/ijerph17155500 32751465 PMC7432647

[B70] Xie X. Wang L. Zeng S. (2018). Inter-organizational knowledge acquisition and firms' radical innovation: a moderated mediation analysis. J. Bus. Res. 90, 295–306. 10.1016/j.jbusres.2018.04.038

[B71] Yang H. Peng C. Du G. Xie B. Cheng J. S. (2023). How does ambidextrous leadership influence technological innovation performance? an empirical study based on high-tech enterprises. Technol. Anal. Strat. Manag. 35, 737–751. 10.1080/09537325.2021.1985105

[B72] Yang S. Ishtiaq M. Anwar M. (2018). Enterprise risk management practices and firm performance, the mediating role of competitive advantage and the moderating role of financial literacy. J. Risk Fin. Manag. 11:35. 10.3390/jrfm11030035

[B73] Zacher H. Rosing K. (2015). Ambidextrous leadership and team innovation. Leaders. Org. Dev. J. 36, 54–68. 10.1108/LODJ-11-2012-0141

[B74] Zahid M. Naeem H. Aftab I. Mughal S. A. (2021). From corporate social responsibility activities to financial performance: role of innovation and competitive advantage. Asia Pacif. J. Innov. Entrepr. 15, 2–13. 10.1108/APJIE-04-2020-004632276480

[B75] Zhang S. Suntrayuth S. (2024). The synergy of ambidextrous leadership, agility, and entrepreneurial orientation to achieve sustainable AI product innovation. Sustainability 16:4248. 10.3390/su16104248

[B76] Zigan K. Macfarlane F. Desombre T. (2007). Intangible resources as performance drivers in European hospitals. Int. J. Product. Perform. Manag. 57, 57–71. 10.1108/17410400810841236

